# Advancing Balance Assessment in Stroke Rehabilitation: A Comparative Exploration of Sensor-Based and Conventional Balance Tests

**DOI:** 10.3390/s26041308

**Published:** 2026-02-18

**Authors:** Marieke Geerars, Natasja C. Wouda, Richard A. W. Felius, Johanna M. A. Visser-Meily, Martijn F. Pisters, Michiel Punt

**Affiliations:** 1 Research Group Lifestyle and Health, Utrecht University of Applied Sciences, 3584 CS Utrecht, The Netherlands; 2 Centre of Excellence for Rehabilitation Medicine, UMC Utrecht Brain Centre, University Medical Centre Utrecht, and De Hoogstraat Rehabilitation, 3583 TM Utrecht, The Netherlands; 3 Axioncontinu, Physiotherapy Department Neurology, Rehabilitation Center de Parkgraaf, Beneluxlaan 926, 3526 KJ Utrecht, The Netherlands; 4 Department of Neurorehabilitation, De Hoogstraat Rehabilitation, Rembrandtkade 10, 3583 TM Utrecht, The Netherlands; 5 Faculty of Human Movement Sciences, Vrije Universiteit Amsterdam, 1081 HV Amsterdam, The Netherlands; 6 Department of Rehabilitation, Physiotherapy Science & Sport, UMC Utrecht Brain Center, Utrecht University, P.O. Box 85500, 3508 GA Utrecht, The Netherlands; 7 Center for Physical Therapy Research and Innovation in Primary Care, Julius Health Care Centers, P.O. Box 85500, 3508 GA Utrecht, The Netherlands; 8 Research Group Empowering Healthy Behavior, Department of Health Innovations and Technology, Fontys University of Applied Sciences, P.O. Box 347, 5600 AA Eindhoven, The Netherlands

**Keywords:** stroke, rehabilitation, balance, inertial measurement unit, IMU, sensor

## Abstract

**Highlights:**

**What are the main findings?**

- Postural sway measures are less prone to floor and ceiling effects and may be well suited for monitoring balance progression in the future.
- Postural sway measurement provides complementary balance information: IMU stance-tasks correlated moderately with the BBS and Mini-BESTest, while IMU sitting-tasks showed weak to no association with the TCT.

**What are the implications of the main findings?**

- IMUs capture balance information that is partially distinct from conventional tests and therefore cannot replace conventional balance tests.
- The clinical value of postural sway measurement in clinical stroke rehabilitation requires further investigation.

**Abstract:**

Balance impairments in stroke rehabilitation are commonly assessed using the Trunk Control Test (TCT), Berg Balance Scale (BBS), and Mini Balance Evaluation System Test (Mini-BESTest). However, these conventional tests are subjective, susceptible to floor and ceiling effects, and time-intensive. Inertial measurement units (IMUs) may address these limitations by providing objective, impairment-level metrics, not captured by conventional tests. This observational study explored the measurement properties of an IMU-based balance assessment of postural sway, and compared them with conventional tests in routine stroke rehabilitation. Stroke survivors from five Dutch rehabilitation centers were assessed at admission and discharge using conventional and IMU-based balance tests during sitting and standing tasks. Floor and ceiling effects were evaluated, and relationships between measures were examined using correlation analysis. At admission, 105 participants were measured, and 90 at discharge. IMU measures showed no floor or ceiling effects despite skewed distributions. IMU stance-tasks correlated moderately with the BBS and Mini-BESTest (18–29% variance explained), whereas IMU sitting-tasks showed weak to no relationship with the TCT. IMU-based balance assessment of postural sway captures balance-related information that is partially different from conventional tests. Although IMUs offer practical advantages, further research is needed to establish the clinical relevance of postural sway measurements alongside conventional tests.

## 1. Introduction

After a stroke, balance disorders are of particular concern, as they can lead to dependency in activities of daily living (ADL), difficulties with ambulation, and falls [1]. Additionally, fear of falling may cause stroke survivors to become inactive, which can negatively affect long-term health [2,3]. Sitting and standing balance are important predictors of functional recovery and hospital stay after an acute stroke [4,5]. Moreover, many post-stroke decisions, such as the need for inpatient rehabilitation and the ability to function safely at home after rehabilitation, are influenced by the individuals’ balance. Therefore, balance assessment is a key aspect in stroke rehabilitation.

The Trunk Control Test (TCT) and the Berg Balance Scale (BBS) are widely used and recommended by guidelines to assess sitting and standing balance [6,7,8,9], but have limitations, including subjectivity—because each item is scored by the physiotherapist—and susceptibility to floor and ceiling effects [7,10,11]. This suggests that both the TCT and BBS may not detect changes in patients with severe balance disorders or those with only mild impairments, potentially overlooking significant progress [8]. To cover the ceiling effect of the BBS in stroke survivors with mild impairments, additional tests like the Mini Balance Evaluations System Test (Mini-BESTest) are recommended [8,11]. This test assesses both static and dynamic balance while exhibiting no ceiling effects [6,12]. Nevertheless, in clinical stroke practice, the Mini-BESTest is sometimes considered infeasible due to time constraints or, in some cases, because it cannot be safely conducted by a single therapist [13]. To overcome these issues, we explored an additive—or potentially alternative—approach to balance assessment, using a body-worn ‘inertial measurement unit’ (IMU). This approach could reduce the time and burden of balance assessment, allow for more frequent application, and minimize floor and ceiling effects compared to conventional tests [14]. Additionally, an IMU can provide precise impairment-level metrics that may characterize how and why balance is impaired and detect small changes that may not be captured by conventional tests. This information might help physiotherapists in better targeting their therapeutic interventions. While previous studies investigated balance using IMUs or technologies such as force plates in laboratory-based setups [15,16,17,18], our study uniquely focused on exploring the measurement properties of IMU scores within the context of routine stroke rehabilitation, providing insights into their application in real-world rehabilitation settings.

Postural control is a complex skill that emerges from sensorimotor, cognitive, and musculoskeletal interactions, providing a state of balance during any posture or activity [19]. Subtle fluctuations of the body during the upright position—referred to as ‘postural sway’—reflect the continuous regulation of these interactions. As such, postural sway provides a sensitive and physiologically meaningful indicator for postural stability and motor control [14,20,21]. Previous research has demonstrated that postural sway can be measured with good reliability and reproducibility across repeated assessments and measurement systems, supporting their use as an objective biomarker for postural control [22,23,24]. Moreover, sway characteristics change with pathology and have shown to differ across ageing and neurological populations, including stroke survivors [25]. Evidence suggests that stroke survivors have increased postural sway compared to healthy controls [16] and numerous studies have shown that increased postural sway goes along with reduced balance control [15,16,18,26].

An IMU is a small device that measures movement in six degrees of freedom, by combining three-dimensional linear acceleration and angular velocity [24]. When worn directly on the body, near the patient’s center of mass (CoM), it can approximate CoM movement and thereby quantify postural sway [15,19]. In our previous study, we concluded that IMUs can reliably and objectively measure postural sway with a small minimal detectable change (MDC) in stroke survivors undergoing rehabilitation [24]. The current study aimed to further investigate the measurement properties of the IMU. First, we examined the floor and ceiling effects of both the conventional and IMU tests. Second, we explored the relationship and assessed the proportion of shared variance between the conventional and IMU tests.

## 2. Materials and Methods

### 2.1. Study Participants

Participants were recruited by their physician or physiotherapist in five inpatient rehabilitation centers in the Netherlands between January 2020 and December 2022. To be eligible, potential participants had to meet the following criteria: (1) a diagnosis of stroke according to the World Health Organization definition [27]; (2) age ≥ 18 years; (3) the ability to sit unsupported for at least one minute; and (4) the ability to understand and perform simple tasks. All participants provided written informed consent.

### 2.2. Design

An observational study was conducted, with data collected at admission and discharge to explore potential floor and ceiling effects. Floor effects were anticipated at admission due to severe impairments in some patients, while ceiling effects were expected at discharge as balance improved during rehabilitation. Balance was assessed using the TCT, BBS, and a composite ‘IMU balance test,’ with IMU measurements from multiple tasks analyzed individually; the Mini-BESTest was administered if the BBS score was ≥45. Conventional tests were conducted by experienced physiotherapists, while the IMU measurements were performed by researchers or trained physiotherapists. Demographic data (e.g., age, stroke type, functional level) were collected at both time points. Patients were enrolled upon eligibility, and if a participant could not sit independently or was too fatigued for the IMU measurements at admission, data from the first week of IMU testing were used as admission data. The medical ethical review committee of Utrecht approved this study (METC number: 20-462/C).

### 2.3. Sample Size Estimation

We aimed to recruit at least 100 participants, following de Vet et al.’s [28] recommendations for large sample sizes (>100) in validation studies calculating correlation coefficients.

### 2.4. Measurements

#### 2.4.1. IMU Balance Test

The IMU balance test was standardized and consisted of postural sway measurements during two sitting and three standing tasks, each lasting 30–60 s (Table 1). We focused exclusively on static balance, as we believed this would provide insights that complement conventional tests. During the sitting tasks, the IMU was positioned on the upper back (Th8) using a belt, while for the standing tasks, the IMU was repositioned to the lower back (L5). An adjustable treatment table was used for the sitting tasks. Unstable sitting was performed on a round, inflatable, balance cushion (MamboMax, 45 cm, semi-soft) placed on the treatment table. For unstable-stance, the participant stood on a foam balance pad (Airex balance pad, dimensions: 50 × 41 × 6 cm).

Participants could rest between tasks and were allowed up to two additional attempts if balance was lost. To isolate postural sway, they were instructed to remain as still as possible and avoid talking during the tasks. Before each task, participants were briefed on instructions and asked if they were ready. The IMU was activated, and the stopwatch started. During the task, participants were updated on the remaining time (‘half a minute to go’, ‘a quarter minute to go’). For eyes closed task, the 30 s were counted aloud, to prevent talking. At the end, the IMU was switched off before participants were informed the task was complete, ensuring they remained still until the recording stopped.

The IMU consisted of a triaxial accelerometer and a gyroscope (manufactured by Aemics B.V., Oldenzaal, The Netherlands) and recorded data at a sampling rate of 104 samples per second. The accelerometer and gyroscope ranges were set to 4 g and 500 degree/s, respectively. For each IMU task, the IMU time series were converted into IMU features [24]. To obtain a summary measure of postural sway, extracted features from each balance task were reduced using a principal component analysis (PCA). PCA was applied separately for each task, resulting in a task-specific score per individual. These scores were intended for interpretation within each task only and were not used to derive a composite outcome score across all five tasks. For further details, see Appendix A. The resulting PCA scores were z-transformed, to reflect the standard deviations (SD) from the mean, providing a fixed range necessary for assessing potential floor and ceiling effects of the IMU.

#### 2.4.2. Trunk Control Test

The TCT evaluates trunk motor performance [29]. It includes three movement items and one balance item (unsupported sitting on the side of a bed). Each item is scored on a three-point ordinal scale (0, 12, or 25), resulting in a total score ranging from 0–100. Higher scores indicate better performance.

#### 2.4.3. Berg Balance Scale

The BBS is a 14-item scale with predetermined tasks to assess static and dynamic balance during sitting and standing [30]. Each item is scored on a five-point ordinal scale from 0 to 4 (0 = unable, 4 = normal). The score ranges from 0–56, with higher scores indicating better balance ability. Assistive devices are not permitted during the test. The BBS is a reliable and valid tool to assess balance in people after stroke [8].

#### 2.4.4. Mini-BESTest

The Mini-BESTest evaluates four systems of balance control: anticipatory balance, reactive balance, sensory orientation, and balance during gait [31]. It comprises 14 items, each scored on a three-point ordinal scale (0 = unable, 1 = moderate, 2 = normal), with a maximum score of 28. Higher scores indicate better balance. The Mini-BESTest is a reliable and valid tool for assessing balance in middle aged and older adults in the subacute phase after stroke who were able to walk [32].

### 2.5. Analysis

All statistical analyses were performed using Statistical Package for the Social Sciences (SPSS) version 27. The significance level was set on *p* ≤ 0.05.

#### 2.5.1. Floor and Ceiling Effects

The score distributions, skewness (ϒ), and kurtosis of all tests (TCT, BBS, Mini-BESTest and IMU) were assessed. Skewness greater that +1 indicates a floor effect, while less than −1 suggests a ceiling effect [33]. Kurtosis was examined to determine whether the data had heavy or light tails compared to a normal distribution. A normal distribution has a kurtosis of 3. Kurtosis increases with peakedness, reflecting heavy tails, and decreases with flatness, reflecting light tails [34]. To further explore floor and ceiling effects, the proportion of participants reaching the maximum score of the TCT, BBS, or Mini-BESTest was examined. Additionally, the proportion reaching the top or bottom 10% scores on the BBS, Mini-BESTest or IMU were calculated. For the BBS (score range 0–56), the top 10% score is >50 and the bottom 10% score is <6. For the Mini-BESTest (score range 0–28), the top 10% score is >25 and the bottom 10% score is <3 [35].

Given the IMU’s infinite range, we selected a specific approach to establish cutoff scores for identifying participants in the top and bottom 10% scores. The IMU data showed a peaked distribution with a few extreme values at the ends, but no clustering at the edges, suggesting that floor and ceiling effects were unlikely [36]. Based on these observations, we included only IMU scores within the range of ±3 (i.e., standard deviations (SD)), capturing 99.7% of the data in a normal distribution. The top and bottom 10% scores of the IMU were calculated by taking 10% of the 6 point range (0.6). The proportion of participants in the top 10% (between −3 and −2.4) and bottom 10% (between 2.4 and 3) was then determined. A proportion greater than 20% in these ranges or at the maximum score indicated a substantial floor or ceiling effect [33].

#### 2.5.2. Relationship and Extent of Covariance Between the IMU and Conventional Tests

Spearman’s Rank correlation coefficients were calculated between the IMU and the TCT, BBS, and Mini-BESTest to explore the strength of the relationship among these tests. We assessed the relationship between the TCT and IMU sitting tasks, as well as between the BBS and IMU standing tasks with EO and EC, as these tests measure comparable aspects of balance. The Mini-BESTest was compared with the IMU unstable-stance task, as both are considered more challenging, which may make them more discriminative for patients with higher balance performance. R^2^ was calculated to establish the proportion of variance shared between the tests, providing insight in the extent of covariance. Correlation coefficients were considered as follows: <0.2 = very weak or no relationship, 0.2–0.4 = weak, 0.4–0.6 = moderate, 0.6–0.8 = strong, and 0.8–1.0 = very strong [37].

## 3. Results

We included a heterogeneous group of stroke survivors with a broad range in age, time since stroke, stroke severity, physical functioning, and ADL independence, providing a representative sample for evaluating balance and postural sway. Detailed demographic and clinical characteristics of the participants are summarized in Table 2. At admission, 105 participants were enrolled. Data from 100 participants were included in the analysis, as IMU measurement errors occurred in five participants. At discharge, 90 participants underwent an IMU test. The 14% dropout was due to various reasons: patients not yet discharged at the study end date (n = 2), insufficient time between measurements because some patients were discharged within the same week as admission, precluding repeat admission (n = 5), other diagnoses (n = 3), death (n = 2), or refusal (n = 3). Data from 81 of the 90 participants were analyzed due to IMU measurement errors.

### 3.1. Floor and Ceiling Effects

**Figure 1:**
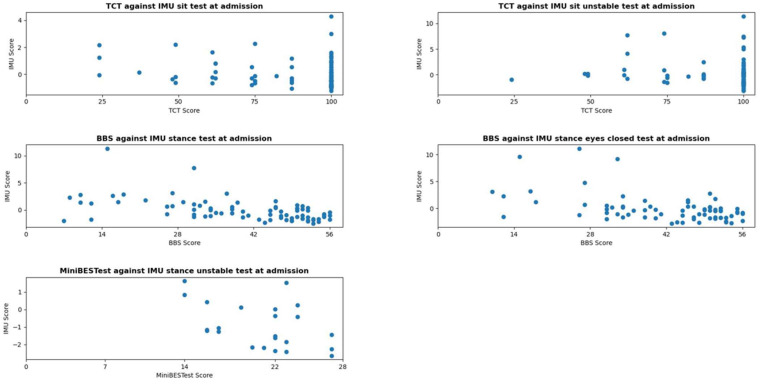
Score distribution and correlation between the sum scores of the conventional balance tests and the IMU-based balance scores at admission. Each dot represents one or more participants, since some participants had equal scores on the clinical measures with similar IMU-scores. Higher IMU-based balance scores indicate larger postural sway, thus reflecting poorer balance.

**Figure 2:**
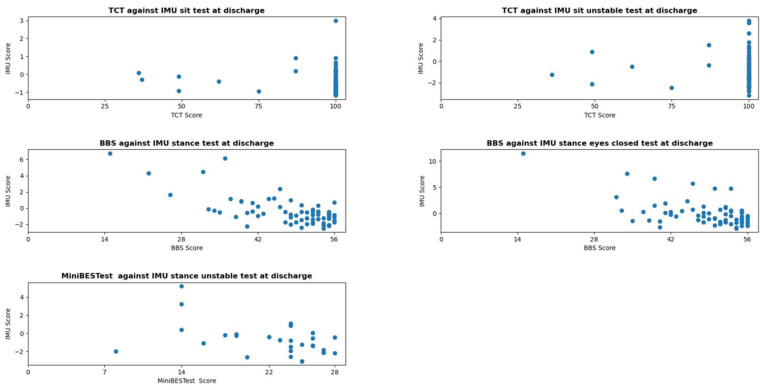
Score distribution and correlation between the sum scores of the conventional balance tests and the IMU-based balance scores at discharge. Each dot represents one or more participants. Higher IMU-based balance scores indicate larger postural sway, thus reflecting poorer balance.

The score distributions of the conventional balance tests and IMU scores are shown in Figure 1 and Figure 2. Table 3 presents the skewness, kurtosis, and potential floor and ceiling effects for these tests at admission and discharge. The conventional test data were negatively skewed, except for the BBS and Mini-BESTest at admission, indicating ceiling effects. The TCT exhibited high kurtosis values, while the BBS and Mini-BESTest at admission showed low kurtosis, indicating a broad distribution. For most conventional tests (except the Mini-BESTest at admission), over 20% of participants reached the top 10% score, suggesting substantial ceiling effects. IMU scores were positively skewed and exhibited high kurtosis, indicating that data peaked around a central value, and few extreme scores. However, no floor or ceiling effects were observed, as fewer than 4% of participants reached the top or bottom 10% of scores.

### 3.2. Relationship and Extent of Covariance Between IMU Measurements and Conventional Tests

The relationship between the IMU measurements and conventional tests is shown in Table 4, and Figure 1 and Figure 2. A weak to no relationship was found between the TCT and IMU sitting-tasks. Moderate negative correlations were observed between the BBS, Mini-BESTest and IMU. For the IMU stance EO task, R^2^ values of 0.29 at admission and 0.28 at discharge indicated that 29% and 28% of BBS score variance were explained by the IMU stance EO task. For the IMU stance EC task, R^2^ values of 0.18 at both measurement moments indicated that 18% of BBS variance was explained. The relationship between the IMU unstable-stance task and the Mini-BESTest yielded R^2^ values of 0.20 at admission and 0.19 at discharge, explaining 20% and 19% of Mini-BESTest variance.

## 4. Discussion

In this study, we explored balance assessment using a body-worn IMU to measure postural sway, compared to conventional balance tests (TCT, BBS, and Mini-BESTest) in stroke survivors undergoing rehabilitation. We examined floor and ceiling effects and investigated the relationship between the tests and the extent of their covariance. Our findings indicate that IMU-based balance assessment is less prone to floor and ceiling effects compared to conventional tests. IMU-based scores were only partially related to stroke survivors’ performance on conventional tests, suggesting that IMU-based balance assessment captures aspects of balance not fully represented by commonly used clinical tests and may therefore provide additional clinically useful information. The differences reflect fundamental distinctions in the constructs assessed by the two approaches. Postural sway reflects the continuous interaction of sensorimotor, cognitive, and musculoskeletal systems [19] and quantitative sway analysis may provide information in how balance is controlled rather than solely whether functional balance tasks can be performed successfully. IMU-based balance scores revealed a wide distribution of postural sway among stroke survivors: some individuals maintained balance with minimal sway, while others required increased micromovements and corrective body movements to remain stable—differences that are not necessarily captured by the conventional tests. Within the International Classification of Functioning, Disability and Health (ICF) framework [38,39], IMU-based measures primarily reflect impairments at the Body Functions and Structures level and are related to underlying sensorimotor control mechanisms [19]. Conventional tests, on the other hand, focus on the performance of functional balance tasks, aligning with ICF’s activity level. As a result, a limited correlation between both assessment methods is not unexpected. Subtle impairments in postural control detected by IMUs may not be severe enough to affect conventional test performance or may be overlooked entirely. In addition, stroke survivors may use compensatory strategies that enable them to achieve high scores on conventional tests, despite persistent impairments captured by the IMU. Additionally, postural sway was measured statically, whereas conventional tests include both static and dynamic tasks, further contributing to the observed differences between both approaches.

The reduced floor and ceiling effects we observed for IMU-based measures are consistent with previous studies in neurological, geriatric, and healthy populations, demonstrating that these measures provide detailed information on postural control not captured by clinical tests [40]. Our findings regarding the limited correlations between IMU-based and conventional tests are consistent with those of Cho et al. [15] who concluded that a decrease in postural sway does not necessarily indicate improved balance, and with Nes et al. [41], who found no relationship between postural sway and the TCT in sitting balance recovery. For stance conditions, we compared our findings with studies by Cho et al. [15] and Sawacha et al. [42], who investigated postural sway in chronic stroke survivors using a force platform. Although force platforms and IMUs are based on different measurement principles, both approaches assess the same underlying construct of postural sway and therefore provide relevant contextual comparisons. As participants were instructed to remain static throughout the IMU-balance tasks, an IMU placed near the body’s center of mass should, in principle, reflect information broadly comparable to center of pressure outcomes derived from force plates. In contrast to our findings, Cho et al. [15] reported no significant correlation between postural sway during stable stance and the BBS, while Sawacha et al. [42] found various correlations between sway features and the BBS in stable stance conditions with EO and EC. However, given the small sample sizes in these studies, their findings should be interpreted with caution [28].

### 4.1. Study Limitations

This study has some limitations. First, because it focused exclusively on stroke survivors in inpatient rehabilitation, the findings may not be generalizable to the broader stroke population. Second, the use of convenience sampling introduces potential self-selection bias, affecting sample representativeness. Additionally, time constraints prevented the inclusion of all eligible patients, which may have influenced the sample composition. Despite this, we included a large group of stroke survivors, with a broad range of stroke severity, balance, mobility, and age. Third, we assessed only static balance with the IMU, excluding dynamic tasks, which may have narrowed our findings. However, static postural sway alone reflects the integration of central balance control mechanisms and can discriminate between healthy and neurologically impaired populations, thereby providing insight into underlying impairments [14,43]. Although static and dynamic assessments capture partly different aspects of balance, our decision to assess only static balance with the IMU reflects typical clinical practice. During dynamic balance tasks, physiotherapists generally assess performance directly to judge fall risk and ensure safe mobility, basing clinical decision-making on their observation and impression rather than relying on IMU scores. Another limitation is task performance variability, as not all participants could execute every IMU task. Missing data in conventional tests, collected during routine clinical practice, further reduced the sample size, meaning the intended sample size of n = 100 for correlation studies was not met for all tasks [26]. Still, given the relatively large sample size, we expect that the overall results would remain relatively stable. Caution is advised when interpreting results for the Mini-BESTest versus IMU unstable-stance task, due to the limited number of participants. The Mini-BESTest was conducted only in one rehabilitation center and was limited to participants with BBS scores ≥ 45.

### 4.2. Clinical Relevance

This study represents an initial step in exploring the potential of postural sway measurement with a body-worn IMU for assessing balance in stroke rehabilitation. Our findings underscore that IMU-based and conventional tests are not interchangeable, as they capture different aspects of balance. Rather than replacing established conventional tests, IMUs appear to provide complementary information, that may enrich balance evaluation. However, the current clinical utility of IMUs remains uncertain, as the interpretation of IMU data is more complex to interpret, making it unclear how they can be utilized in rehabilitation, to inform decisions and improve care. Future studies should address IMUs sensitivity to change and responsiveness. Additionally, identifying relevant biomarkers for physical functioning and establishing functional cutoff values for clinical decision-making may enhance their clinical relevance. While conventional balance tests are likely to remain important reference measures, incorporating complementary validation criteria—such as fall incidence and participation-level outcomes—may further clarify the added value of IMUs. If IMUs can provide unique and clinically relevant insights, alongside practical advantages—being objective, quick, easy to apply, and unaffected to floor and ceiling effects—on which therapists can base their decisions, they could become valuable complementary tools in clinical practice.

## 5. Conclusions

Balance assessment using a body-worn IMU to measure postural sway captures partially different information compared to conventional balance tests. Although IMUs offer practical advantages, further research is needed to understand the clinical relevance of postural sway measurements in addition to the conventional tests.

## Figures and Tables

**Table 1:** IMU balance test: Description of tasks measured with the IMU.

IMU Task	Duration	Description	
Stable sit	60 s	Sitting unsupported on a flat, stable underground, feet on the ground on a self-selected distance, hips and knees in a 90° position	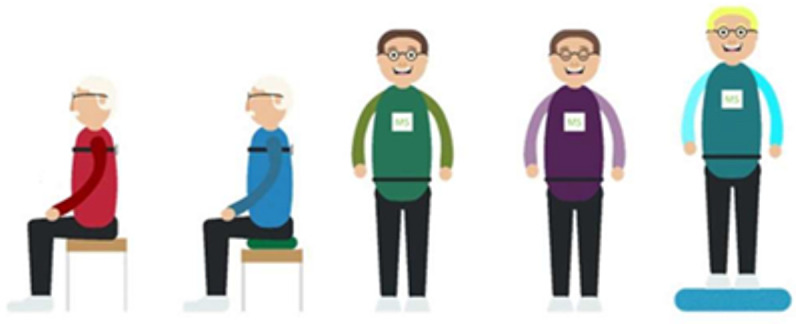
Unstable sit	60 s	Sitting unsupported on an aircushion, placed on a flat, stable underground, feet on the ground, maximally 15 cm apart	
Stable stance EO	60 s	Standing unsupported, self-selected foot position	
Stable stance EC	30 s	Standing unsupported with eyes closed, self-selected foot position	
Unstable stance	30 s	Standing unsupported on a foam cushion, self-selected foot position	

Each task of the IMU balance test was recorded and scored individually using the IMU. IMU, inertial measurement unit; EO, eyes open; EC, eyes closed.

**Table 2:** Participant characteristics.

Characteristics	All Participants Admission (N = 105)	All Participants Discharge (N = 90)
**Demographics**		
Age (years)	72.7 (12.2) [28–99]	72.6 (12.3) [28–99]
Sex (female/male) n (%)	51/54 (48.6/51.4%)	45/45 (50/50%)
Body mass index (kg/m^2^)	26.0 (5.3) [10.3–45.4]	26.3 (5.5) [10.3–45.4]
**Stroke characteristics**		
Ischaemic n (%)	82 (78%)	71 (78.9%)
Haemorrhagic n (%)	18 (17.1%)	16 (17.8%)
SAH n (%)	5 (4.8%)	3 (3.3%)
Location stroke n (%)		
▪cortical	56 (55.0%)	47 (52.2%)
▪subcortical	10 (10.0%)	10 (11.1%)
▪midbrain	7 (7.0%)	6 (6.7%)
▪brainstem	11 (11.0%)	10 (11.1%)
▪undetermined	21 (17.0%)	17 (18.9%)
Time since stroke (weeks)	2.2 (2.2) [0.7–13.7]	8.9 (4.7) [3.0–21.0]
Paretic side n		
▪Left	46	40
▪Right	35	28
▪Both sides	4	3
▪None	20	19
Barthel Index (0–20) (n = 101/77)	13.0 (4.9) [0–20]	17.7 (4.0) [1–20]
MRS (0–5) (n = 72/62)	3.5 (0.8) [2–5]	2.4 (0.9) [1–4]
NIHSS (0–34) (n = 70/59)	4.5 (3.5) [0–18]	1.9 (2.6) [0–13]
**Motor performance**		
MI LE L (0–100) (n = 93/75)	87.1 (22.5) [0–100]	89.4 (19.6) [9–100]
MI LE R (0–100) (n = 92/75)	90.0 (18.6) [0–100]	95.5 (13.0) [34–100]
FMA-LE score (0–34) (n = 47/48)	26.8 (8.8) [4–34]	30.0 (6.7) [8–34]
**Conventional balance test performance**		
TCT (0–100) (n = 103/85)	87.6 (22.8) [12–100]	96.3 (18.1) [36–100]
BBS (0–56) (n = 100/80)	34.7 (17.2) [3–56]	46.1 (11.9) [4–56]
Mini-BESTest (0–28) (n = 23/28)	20.8 (4.0) [14–27]	22.1 (5.1) [8–28]
**IMU balance performance**		
Stable sit (n = 100/80)	−0.29 (−0.67, 0.53)	−0.55 (−0.85, −0.05)
	[−1.23, 4.31]	[−1.18, 6.95]
Unstable sit (n = 93/79)	−0.17 (−1.28, 0.84)	−1.04 (−1.79, 0.06)
	[−3.14, 11.44]	[−3.21, 17.97]
Stable stance EO (n = 84/77)	−0.37 (−1.43, 0.83)	−0.81 (−1.46, 0.27)
	[−2.49, 18.48]	[−2.50, 6.77]
Stable stance EC (n = 75/74)	−0.44 (−1.64, 0.32)	−0.70 (−1.66, 0.52)
	[−2.80, 11.12]	[−2.87, 11.53]
Unstable stance (n = 65/73)	−0.42 (−1.50, 0.42)	−0.44 (−1.46, 0.60)
	[−2.95, 17.20]	[−3.06, 8.52]

Values of the demographics, stroke characteristics, and motor and balance performance on the conventional tests were expressed as mean (SD) and range [min, max] or n and percentage (%). IMU-based balance scores were expressed as median (IQR) and range [min, max], due to the skewness and kurtosis values of the data. SAH, subarachnoid haemorrhage; L, left; R, right; MRS, Modified Ranking Scale; MI LE, Motricity Index Lower Extremity; FMA-LE, Fugl Meyer Assessment-Lower Extremity; TCT, Trunk Control Test; BBS, Berg Balance Scale; Mini-BESTest, Mini Balance Evaluation Systems Test.

**Table 3:** Comparison of all balance tests: Floor and ceiling effects at admission/discharge.

Balance Tests	Skewness (ϒ)	Kurtosis	Floor Effect (% Participants Obtaining the Worst 10% Score)	Ceiling Effect (% Participants Obtaining the Best 10% Score)	Ceiling Effect (% Participants with the Best Possible Score)
Trunk Control Test (n = 98/79)	−1.87/−3.53	2.71/11.65	0/0	**76.1/89.7**	**69.8/89.7**
Berg Balance Scale (n = 97/74)	−0.62/−2.06	−0.96/4.39	10.5/4.1	**22.3/43.3**	3.2/8.1
Mini-BESTest (n = 23/28)	−0.19/−1.15	−0.89/0.69	0/0	13.0/**32.1**	0/7.1
IMU sit stable (n = 100/80)	1.69/4.62	3.79/28.73	0/0	0/0	N/A
IMU sit unstable (n = 93/79)	1.99/5.20	4.81/36.53	3.3/0	0/0	N/A
IMU stance EO (n = 84/77)	3.94/2.01	20.7/4.74	1.2/4	0/0	N/A
IMU stance EC (n = 75/74)	2.45/2.26	7.37/6.44	0/1.4	0/0	N/A
IMU stance unstable (n = 65/73)	3.80/2.15	19.92/5.81	0/0	0/0	N/A

Mini-BESTest: Mini Balance Evaluation Systems Test; IMU, inertial measurement unit; EO, eyes open; EC, eyes closed; N/A, not applicable. **Bold**: Substantial floor/ceiling effect.

**Table 4:** Spearman’s Rank correlation coefficients (r) IMU balance test with conventional tests TCT, BBS, and Mini-BESTest.

IMU Task		Admission			Discharge	
	TCT	BBS	Mini-BESTest	TCT	BBS	Mini-BESTest
Sit	r = −0.23 * p < 0.02 n = 96			r = −0.13 *p* < 0.26 n = 78		
Sit unstable	r = −0.13 *p* < 0.22 n = 89			r = −0.05 *p* < 0.67 n = 77		
Stance EO		r = −0.54 ** p < 0.001 n = 81			r = −0.53 ** p < 0.001 n = 71	
Stance EC		r = −0.42 ** p < 0.001 n = 72			r = −0.42 ** p < 0.001 n = 68	
Stance unstable			r = −0.45 * p < 0.03 n = 23			r = −0.44 * p < 0.02 n = 28

IMU, inertial measurement unit; EC, eyes closed; TCT, Trunk Control Test; BBS, Berg Balance Scale; Mini-BESTest, Mini Balance Evaluation System Test ** correlation is significant at 0.01 level; * correlation is significant at 0.05 level.

## Data Availability

The data supporting the findings of this study are openly available in Data Archiving and Networked Services (DANS) at https://doi.org/10.17026/LS/FH0XRJ.

## Abbreviations

The following abbreviations are used in this manuscript:

TCT	Trunk Control Test
BBS	Berg Balance Scale
Mini-BESTest	Mini Balance Evaluation Systems Test
IMU	Inertial Measurement Unit
ADL	Activities of Daily Living
CoM	Center of Mass
BoS	Base of Support
MDC	Minimal Detectable Change
PCA	Principal Component Analysis
SD	Standard Deviation

## Appendix A

In our previous study [24], we established the reliability of measuring various balance tasks using a body-worn IMU (i.e., sitting stable, sitting unstable, stance stable with eyes open (EO) and eyes closed (EC), and stance unstable). The IMU revealed many features related to the (postural sway) movement that was gathered at the upper/lower back of the stroke survivor. Since all these features quantify postural sway, they may contain redundant information. To address this, a principal component analysis (PCA) was conducted to reduce dimensionality, while retaining maximum variance [44]. Prior to the PCA, the sampling adequacy of all postural sway features was estimated using the Kaiser–Meyer–Olkin measure (KMO). An overall KMO and a per-feature KMO exceeding 0.7 and 0.5, respectively, were considered acceptable for analysis [45]. To evaluate the robustness and reliability of the principal components, we used the test and retest data from the study conducted by Felius et al. [24], which were not utilized in the PCA computation.

For the PCA, we selected 12 postural sway features that exhibited reliability across all IMU balance tasks. The overall KMO of each condition exceeded 0.5, indicating the suitability of conducting the PCA. All conditions resulted in two principal components with eigenvalues greater than 1. For each task, more than 80% of the variance was captured in the two principal components. All principal components were measured with good-excellent reliability (ICC > 0.7) [45]. Table A1 presents the explained variance ratio and the reliability of these principal components. For the current study, we only used PCA1 of each IMU balance task, as this principal component represented the highest explained variance.

**Table A1:** Explained variance and reliability of the principal components.

	Sit		Sit Unstable *		Stance EO		Stance EC		Stance Unstable	
	PCA1	PCA2	PCA1	PCA2	PCA1	PCA2	PCA1	PCA2	PCA1	PCA2
EVR	57.2%	27.6%	55.8%	27.4%	57.8%	26.4%	60.4%	21.3%	64.6%	16.6%
RMSE	0.475	0.526	-	-	0.283	0.811	0.47	0.88	0.475	0.629
ICC [CI]	0.913 [0.84, 0.95]	0.872 [0.77, 0.93]	-	-	0.962 [0.92, 0.98]	0.707 [0.48, 0.84]	0.905 [0.8, 0.96]	0.764 [0.55, 0.88]	0.902 [0.79, 0.96]	0.819 [0.63, 0.92]
MDC (SEM)	0.815 (0.294)	1.297 (0.468)	-	-	1.29 (0.465)	1.288 (0.465)	3.311 (1.194)	1.542 (0.556)	3.453 (1.246)	1.305 (0.471)

An interclass correlation coefficient (ICC) of >0.7 was considered a good reliability, and an ICC of >0.9 an excellent reliability. Principal components with good to excellent reliability were marked in bold. Abbreviations: EVR, explained variance ratio; RMSE, root-mean-square error; ICC, intraclass correlation coefficient; CI, confidence interval; MDC, minimal detectable change; SEM, standard error of the mean. * The Sit unstable task was not included in the test-retest study of Felius et al. [44].
